# *In vitro* single molecule and bulk phase studies reveal the AP-1 transcription factor cFos binds to DNA without its partner cJun

**DOI:** 10.1016/j.jbc.2022.102229

**Published:** 2022-07-01

**Authors:** James T. Leech, Andrew Brennan, Nicola A. Don, Jody M. Mason, Neil M. Kad

**Affiliations:** 1 School of Biosciences, University of Kent, Canterbury, United Kingdom; 2 Department of Biology & Biochemistry, University of Bath, Bath, United Kingdom

**Keywords:** DNA tightropes, fluorescence, transcription factors, AP-1, CD, single-molecule, AP-1, activator protein 1, CD, circular dichroism, CRE, cyclic-AMP response element, FRET, Förster resonance energy transfer, mNG, mNeonGreen, mCH, mCherry, TRE, 12-O-tetradecanoylphorbol-13-acetate response element

## Abstract

The AP-1 transcription factor family crucially regulates progression of the cell cycle, as well as playing roles in proliferation, differentiation, and the stress response. The two best described AP-1 family members, cFos and cJun, are known to dimerize to form a functional AP-1 heterodimer that binds to a consensus response element sequence. Although cJun can also homodimerize and bind to DNA, the canonical view is that cFos cannot bind DNA without heterodimerizing with cJun. Here, we show that cFos can actually bind to DNA in the absence of cJun *in vitro*. Using dual color single molecule imaging of cFos alone, we directly visualize binding to and movement on DNA. Of all these DNA-bound proteins, detailed analysis suggested 30 to 46% were homodimers. Furthermore, we constructed fluorescent protein fusions of cFos and cJun for Förster resonance energy transfer experiments. These constructs indicated complete dimerization of cJun, but although cFos could dimerize, its extent was reduced. Finally, to provide orthogonal confirmation of cFos binding to DNA, we performed bulk-phase circular dichroism experiments that showed clear structural changes in DNA; these were found to be specific to the AP-1 consensus sequence. Taken together, our results clearly show cFos can interact with DNA both as monomers and dimers independently of its archetypal partner, cJun.

Activator protein 1 (AP-1) represents a group of dimeric transcription factors composed of members of the Jun, Fos, and ATF protein families (1). Individual AP-1 proteins possess leucine zipper regions for dimerization and basic regions for DNA binding, a motif defining all bZIP proteins (2, 3). AP-1 proteins also feature transactivation domains which facilitate transcription initiation (1). bZIP proteins can form homodimers and heterodimers, which increases the diversity of function from a limited number of proteins. Functions identified for AP-1 complexes include cell proliferation, differentiation, repair, and response to stress (1, 4, 5, 6, 7). These complexes are involved in immediate-early gene pathways (8), allowing rapid modulation of transcriptional profiles in response to stressors such as viral infection (9). Furthermore, AP-1 complexes have been strongly implicated in the development of cancer (10, 11, 12), with aberrant expression or regulation of AP-1 proteins leading to uncontrolled proliferation and angiogenesis in tumours (13). Therefore, understanding how oncogenic AP-1 binds DNA has significant value for the development of novel cancer therapeutics (14, 15, 16).

The archetypal and most well-studied AP-1 complex is the cFos:cJun heterodimer, which binds and activates transcription at the 12-O-tetradecanoylphorbol-13-acetate response element (TRE), with a 7 bp consensus sequence TGA[C/G]TCA (3, 17). cFos:cJun is also capable of binding the cyclic-AMP response element (CRE), with the 8 bp consensus sequence TGACGTCA with a similar reported affinity (18, 19). These AP-1 binding sites have been largely deselected from the mammalian genome, particularly in coding regions, whereas AP-1 controlled promoters often contain more than one copy of the TRE site (20). In the absence of cFos, cJun has been shown to homodimerize and bind TRE/CRE sites (3, 21, 22) and can also activate transcription (23). The status of cFos as an independent DNA-binding protein is disputed. Several previous studies have suggested that cFos is incapable of homodimerization and DNA binding due to poor interaction dynamics within the leucine zipper, which comprises a number of Thr/Lys residues within the core region typically comprised of hydrophobic residues (3, 16). While isolated cFos leucine zippers have displayed a low affinity/unstable interaction (24), cFos has been defined as a DNA-binding protein and transcription factor only in the presence of cJun (25). However, Kohler & Schepartz (26) determined through a bulk phase kinetic study that prebinding of cJun and cFos to the DNA before dimerization was the preferred mechanism of AP-1 formation, implying that cFos binds DNA independently. Limited *in vivo* evidence also suggests the existence of cFos homodimers (27). Nonetheless, there is still no clear evidence to support the DNA-binding activity of a cFos homodimer.

We have performed a comprehensive study of the nature and prevalence of the DNA bound forms of cJun and cFos. By fluorescently tagging the bZIP domains of the AP-1 proteins cJun and cFos with different colors and visualizing their interactions on DNA tightropes (single DNA molecules suspended between surface pedestals) at the single molecule level, we found cJun primarily formed homodimers and was able to heterodimerize with cFos as expected. Unexpectedly, however, cFos was found to bind, as a mixture of monomers and dimers, to DNA tightropes in the absence of cJun, which was confirmed using bulk phase circular dichroism (CD) studies. cFos dimerization was further observed in a Förster resonance energy transfer (FRET) assay using fluorescent protein fusions in which either half of the homodimer population was uniquely tagged. Altogether, these observations provide compelling evidence that cFos has a cJun-independent interaction with DNA.

## Results

### AP-1 proteins bind to and diffuse on DNA tightropes

**Figure 1:**
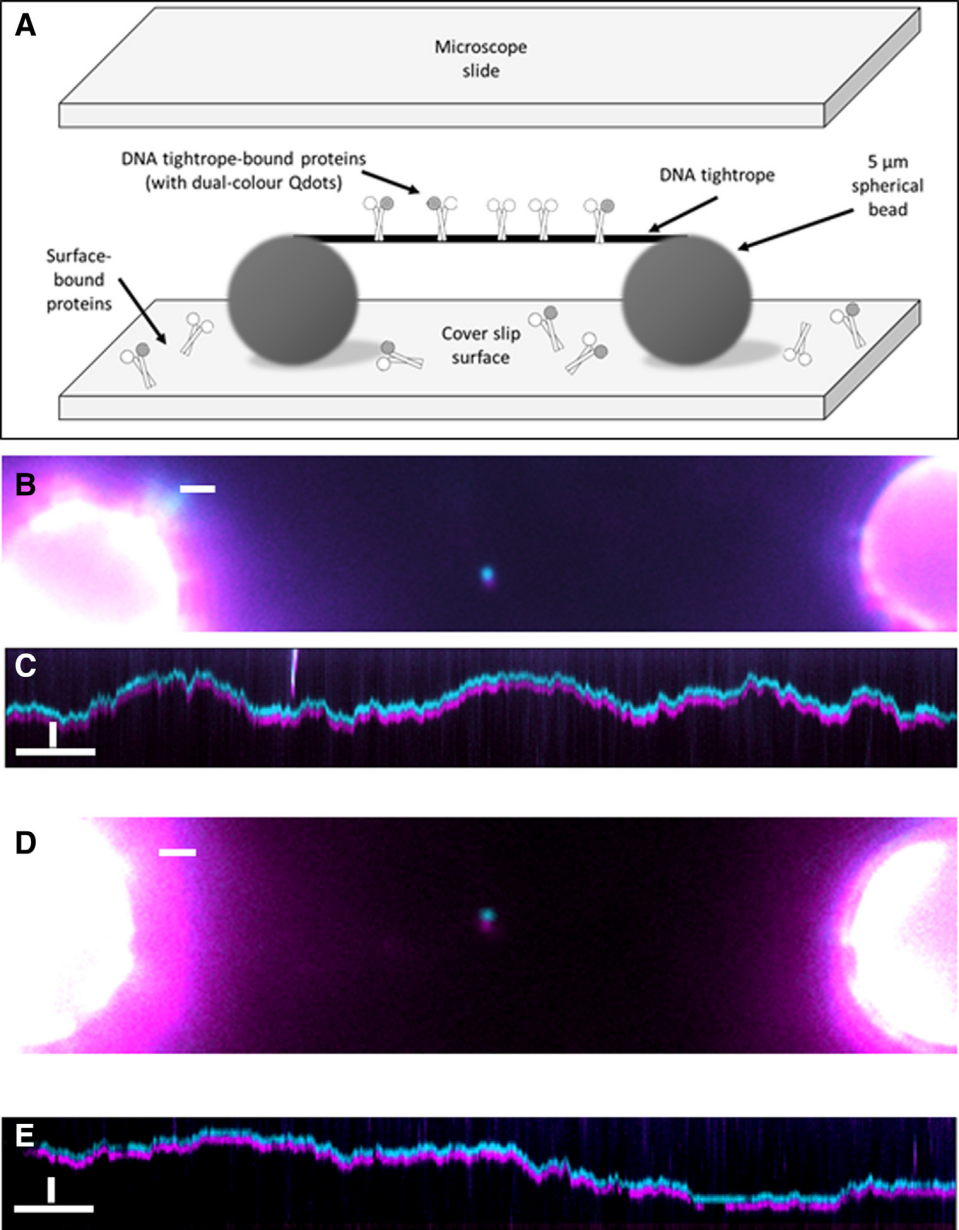
**Imaging AP-1 interactions with DNA using tightropes***. A*, diagram of a DNA tightrope bound with AP-1 proteins and suspended between two surface adhered glass beads. *B*, dual color image of a cJun:cJun homodimer showing colocalization of Qdots. *C*, a kymographic representation of cJun:cJun homodimer position though time, showing clear diffusion on the tightrope. *D*, dual color image of a cFos:cFos homodimer bound to a DNA tightrope. *E*, both Qdots are seen to diffuse on the DNA confirming the existence of a cFos:cFos homodimer. Scale bars in images = 1 μm. Scale bars in kymographs = 5 s (horizontal) *versus* 1 μm (vertical). Videos to accompany these images are included in the supplementary information. AP-1, activator protein 1.

To study the DNA binding and search mechanisms of AP-1 proteins, we used chemically synthesized peptides of the bZIP regions of cJun and cFos. These were modified with a c-terminal biotin tag to allow conjugation with streptavidin-coated Qdots which provide bright and photostable fluorescence emission. Single DNA molecules suspended between surface-immobilized beads (DNA tightropes—Fig. 1*A*) were used as an imaging substrate. This architecture enables high signal to noise imaging with the molecules suspended microns above the surface and therefore free of surface-induced artifacts such as binding to the coverslip surface (28). We used single color– or dual color–labeled cJun or cFos in these experiments, and the proteins were not mixed together in this work as we investigate homodimerization only. The images in Figure 1, *B* and *D* show examples of dual-colored homodimers bound to DNA. These molecules diffused on the DNA in a random walk (see supporting videos), to represent that motion we used kymographs (Fig. 1, *C* and *E*), which are projections of each position (y-axis) over time (x-axis).

### Determining the stoichiometry of cJun and cFos binding to DNA using dual color labeling

**Figure 2:**
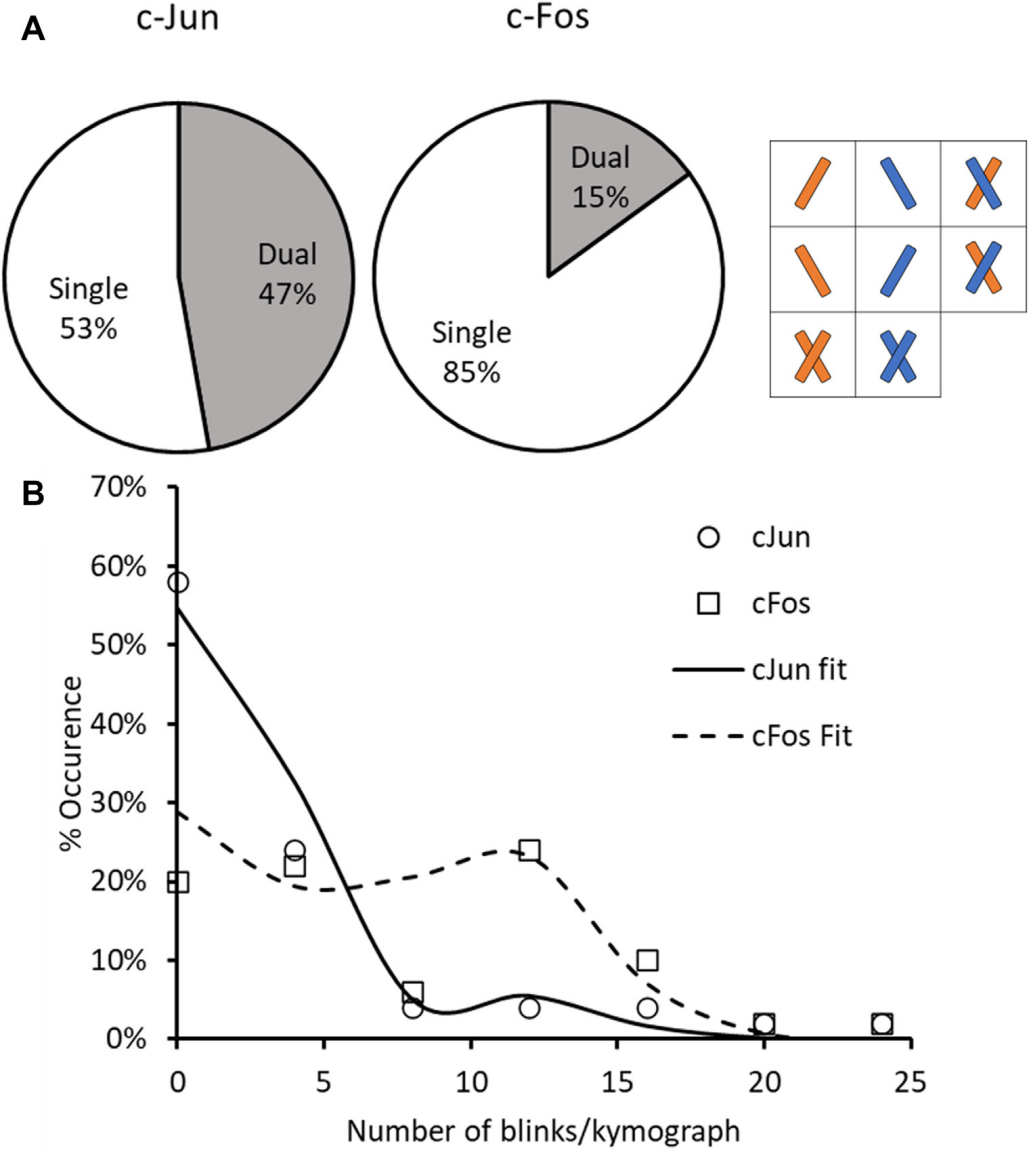
**Determining the oligomeric state of cJun and cFos***. A*, summary of dual color observations of single fluorescent spots on DNA tightropes, on the right is a breakdown of the possible combinations of dual-colored proteins. *B*, histogram of the percentage occurrence of quantum dot blinks per kymograph for cJun (*circles*) and cFos (*squares*). These data were fitted with a combined Poisson relationship (R^2^ = 0.89), and the expected values derived were 1.94 and 10.8 blinks/kymograph (dimer and monomer respectively). cFos fitted to 45.8% ± 6.7 dimer (*dashed line*) and cJun to 87.1% ± 6.9 (*solid line*). All kymographs were identical in duration (60 s), n = 50 for each protein, five flowcells for cJun and nine flowcells for cFos.

From the images in Figure 1, *D* and *E*, it is clear that cFos binds to DNA independently of cJun. Using dual color labeling, we investigated the oligomeric state of cFos when bound to DNA. Equimolar Qdot 655 and Qdot 605 were mixed prior to cFos conjugation to allow an equal probability of a protein binding to either colored Qdot. Whenever molecules dimerize, the color combinations include dimers formed from only 605-labeled cFos, only 655-labeled cFos, and dual-colored cFos (there are two ways dual-colored complexes are formed; therefore, the probability of these forming is twice that of the singly colored entities, see Fig. 2*A*). Due to this, the quantity of dual-colored molecules always underestimates the total number of dimers by 50%. As a consequence, if 100% of molecules dimerized, only 50% of the observed bound molecules would be dual colored. In the case of cFos, we observed 15% ± 1.9 dual-colored entities bound to DNA, indicating that ∼30% of all DNA-bound molecules had dimerized. To ensure that this was not an artefact of labeling, we also studied the occurrence of dual color signals for cJun and found 47% ± 1.3 were dual colored, consistent with nearly complete homodimer formation (Fig. 2*A*). Furthermore, this also indicated that labeling was efficient, since lower efficiency would lead to overrepresentation of singly colored species.

### Using Qdot blinking to determine oligomeric state

The surprising result that cFos binds as a monomer was tested using Qdot blinking. Due to the photophysical properties of Qdots, it is not possible to relate their intensity to the number of Qdots present (29). Therefore, an alternative method to determine oligomeric states was devised based on the blinking of the attached Qdots. The analysis is simple: the chance of a molecule dropping to a completely dark state will be reduced if there are two Qdots present, since the probability of blinking at the same time is the square of that from a single Qdot, resulting in ‘fluorescence redundancy’. The number of blink events was calculated from 50 kymographs (using Qdot 655 conjugates only) and displayed as a histogram (Fig. 2*B*). Fifty-eight percent of cJun kymographs exhibited between 0 and 4 blinks, compared to 20% for cFos. The histogram also displays a prominent peak between 9 and 12 blinks for cFos but a much smaller peak for cJun. This implies the presence of two cFos populations: dimers with few blinks and monomers with a greater number of blinks. A simple Poisson distribution for a single, monomeric species would not reproduce the data; therefore the data in Figure 2*B* were fitted to a dual Poisson model for stochastic blinking. This model assumes that the probability of blinking for dimers is the square of that for monomers. Therefore, only the monomer blinking probability and the amplitude of the monomer population is fitted (see methods); both cJun and cFos were simultaneously fitted, reducing the degrees of freedom. The excellent fit to the data (lines in Fig. 2*B*) validates the model choice, and the amplitudes for the two populations reveal remarkable similarity to that from the dual color experiment; 46% of cFos molecules were dimers compared to 87% for cJun.

### Investigation of cJun and cFos dimerization using FRET

**Figure 3:**
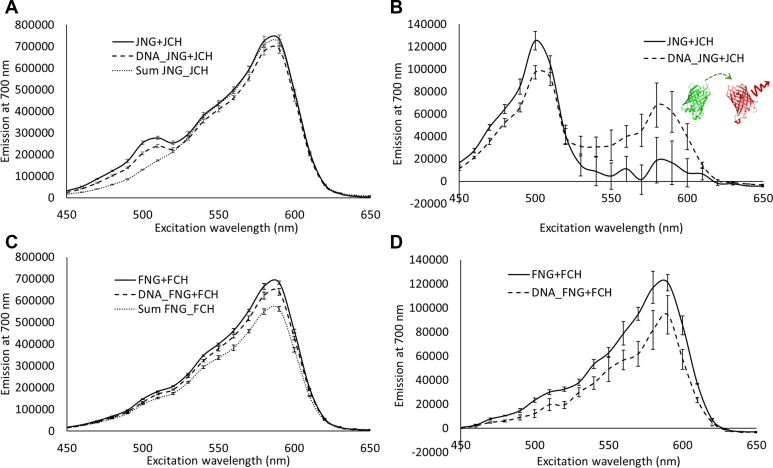
**FRET studies indicate the formation of cJun and cFos homodimers***. A*, excitation spectra of cJun-mNeonGreen paired with cJun-mCherry (JNG + JCH) and also with DNA (DNA_JNG + JCH). The excitation spectrum of the linear sum of the individual proteins is also shown (Sum JNG_JCH). The protein spectra are subtracted from the Sum JNG_JCH reference excitation spectrum to generate (*B*) difference spectra. These show the strongly enhanced contribution of mNG to the emission of mCH. The inset is a cartoon representation of the mechanism of energy transfer from mNG to mCH drawn using PDBs 5LTP and 6YLM. *C*, shows excitation spectra for cFos-mNeonGreen paired with cFos-mCherry (FNG + FCH) and also with DNA (DNA_FNG + FCH). *D*, difference spectra created by subtraction from Sum FNG_FCH in (*C*) reveal an enhancement of the mCH component of the excitation spectra. These data are the mean averages of excitation scans performed in triplicate using a fixed emission wavelength of 700 nm. Error bars represent the standard error of the mean. See Figs. S1–S9 for a more detailed analysis. Elements of these figures are reproduced in the supplementary figures. Fig. S10 shows that the mixing of two noninteracting partners (cFos-mCH and UvrA-mNG) do not exhibit significant energy transfer effects. FRET, Förster resonance energy transfer; mNG, mNeonGreen; mCH, mCherry.

The use of FRET to detect AP-1 interactions is well established but has been primarily used to study cFos:cJun complexes (26, 27, 30, 31). The use of Qdots was ideal for single molecule measurements, but their use in FRET is complicated by surface conjugations and their broad excitation spectra. Therefore, we investigated cJun and cFos homodimerization in solution by exploiting the established FRET pair (32) mNeonGreen (mNG) and mCherry (mCH) fused to cJun or cFos (cJun-mNG, cJun-mCH, cFos-mNG, and cFos-mCH) in a 96-well plate-based fluorescence assay. We generated excitation spectra detecting mCherry emission at 700 nM across an excitation range from 450 to 650 nm. This approach provides an excellent means to detect FRET without bleed-through complications. For cJun (Fig. 3*A*), a clear mNG contribution to the fluorescence at 700 nm is seen. When subtracted from the sum of the individual cJun-mNG and cJun-mCH spectra, a very clear excitation peak for mNG was seen, as predicted when the two fluorophores are coupled due to dimerization (Fig. 3*B*). By comparison, cFos did not have a significant peak at the excitation of mNG (Fig. 3*C*), and the difference spectra (Fig. 3*D*) revealed an increase in fluorescence at 590 nm, consistent with the excitation of mCH only.

These FRET data clearly indicate that a cJun homodimer is forming, and that in the presence of DNA, a conformational change in the homodimer leads to a small increase in mCH excitation. For cFos, the situation is more complex, and the difference spectra show a small contribution from mNG, indicating FRET is occurring for this dimer, though significantly less than for cJun. However, a clear enhancement of fluorescence at 590 nm is present only in the paired combination relative to the sum of their individual spectra, indicating that the change in mCH excitation is only present when cFos-mNG and cFos-mCH are combined *in vitro*. Therefore, these data indicate that cFos forms dimers both with and without DNA present. A more detailed explanation of the FRET spectral analysis is included in the Figs. S1–S9. Fig. S10 shows that noninteracting protein fusions (cFos-mCH and UvrA-mNG) do not elicit energy transfer effects indicating that the effects seen for cFos homodimers are significant and specific.

### CD detection of cFos binding to DNA

**Figure 4:**
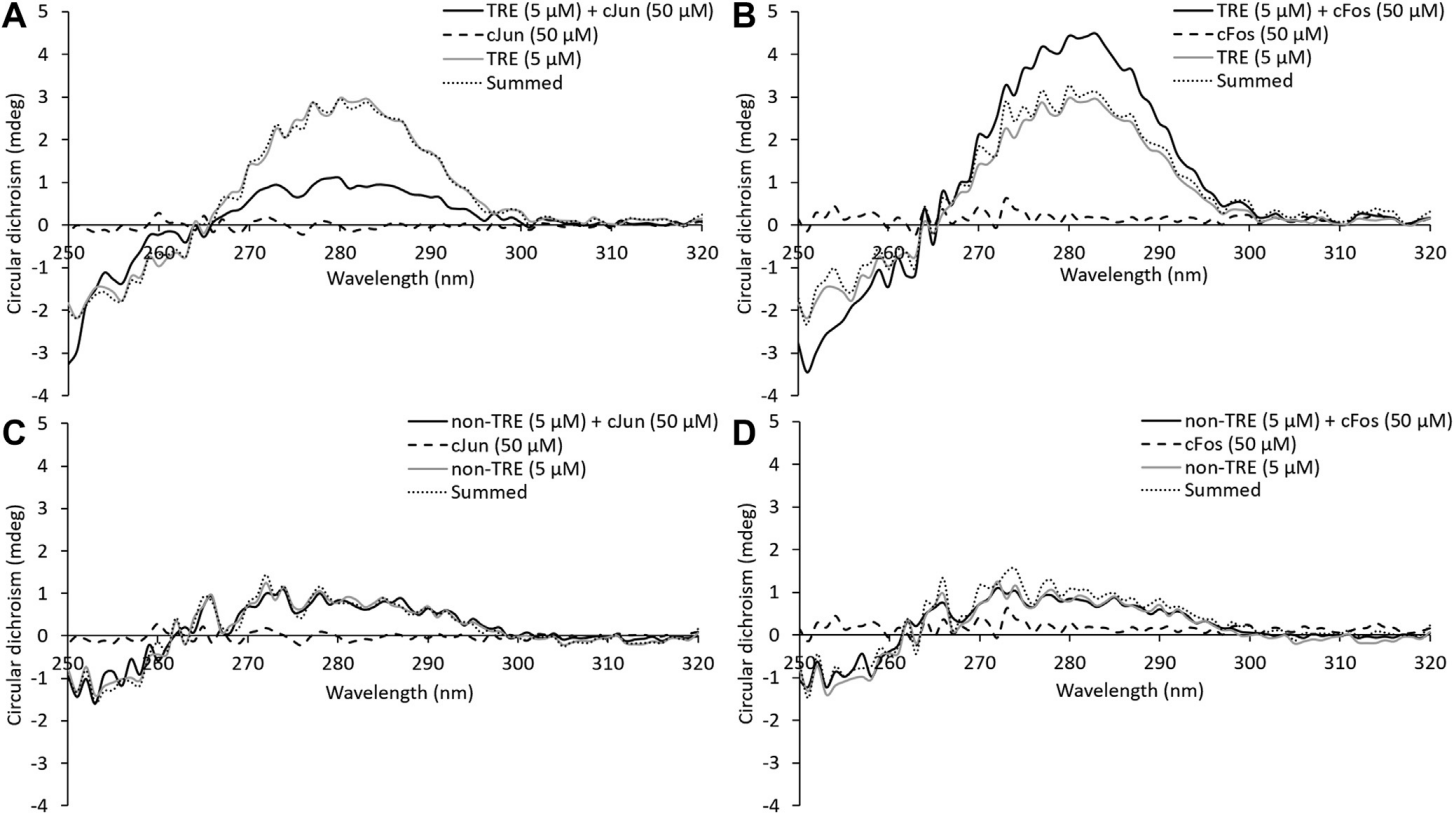
**Far CD spectra indicate TRE DNA bind to cJun and cFos**. The measured spectrum of TRE and (*A*) cJun or (*B*) cFos at 10-fold excess does not overlay with the sum of the individual spectra, indicating a change in structure of the components upon interaction. This change in amplitude of the peak centered on 281 nm is not observed for (*C*) cJun and (*D*) cFos when mixed with non-TRE DNA, indicating that the binding is sequence specific. CD, circular dichroism; TRE, 12-O-tetradecanoylphorbol-13-acetate response element.

To provide an orthogonal approach to our fluorescence observations, we measured the binding-induced structural changes of short oligonucleotides using CD (33). Synthesized AP-1 peptides have previously been explored by Mason et al. (16) and show similar spectra to the biotinylated peptides used in this study. Each component was measured individually, and the sum of the spectra predicts the spectrum for the mixture in the absence of any interaction. The differences observed between the summed spectrum and the measured protein/DNA mixture spectrum provide information on binding (34). Upon addition of 10-fold excess of cFos or cJun to TRE DNA (Fig. 4, *A* and *B*), the amplitude of the peak centered on 281 nm is altered. This indicates binding and reflects changes in the DNA component. Addition of cFos increases the amplitude, whereas cJun decreases it, perhaps indicating different binding modes but clearly showing a change in DNA structure upon protein binding in both cases. As a control, a non-TRE–containing oligonucleotide was used, and no change relative to the summed amplitude was observed (Fig. 4, *C* and *D*). These data indicate clear and specific binding to the TRE consensus sequence.

## Discussion

AP-1 proteins form an array of potential interactors multiplying their possible effects through dimerization. cFos is known to form a heterodimer with cJun and initiate transcription of an array of genes, many of which are oncogenes. However, the role of cFos alone as an entity capable of interacting with DNA has not been considered significant. This stems from a number of studies which have failed to show any dimer formation. However, one study has suggested that cFos can dimerize *in vivo* (27), but *in vitro* support for this view is limited. By using a single molecule imaging approach supported by bulk phase FRET and CD observations, we have determined that cFos associates with DNA both as monomers and dimers. Using cJun, which has a known propensity to homodimerize as a control, we find the equilibrium towards monomer is favored for cFos, and only 30 to 46% of molecules observed on DNA were dimeric. These observations imply that cFos possesses DNA binding activity in the absence of cJun, which could potentially have cellular significance. Given the protooncogenic role of cJun:cFos, this presents an important new area of research for this protein.

In this study, it was imperative to provide a number of approaches to show cFos binds to DNA. We firstly used single molecule imaging of dual-colored complexes. This showed ∼30% of cFos molecules form dimers on DNA compared to 94% for cJun. Importantly, dual-colored cFos was observed to undertake a random walk on DNA, since both colors followed the same path, this provides very strong evidence that their colocalization occurred through complex formation. Numerous proteins undergo random walks when bound to DNA, and this has been suggested to underpin the faster than diffusion target location for proteins such as LacI (35, 36). It is possible that cFos and cJun use one-dimensional diffusion for target site location, and we are currently investigating this possibility. However, to provide additional evidence for homodimer formation, we also measured the frequency of blinking. In these experiments, two molecules are unlikely to blink at the same time so the number of blinks to background should reduce when dimerized. A similar approach has been employed to study the formation of oligomeric complexes on cell surfaces (37). Our approach differs in the statistics we use, since we are measuring blinks for a specific interval, a Poisson rather than binomial distribution is more appropriate. From this analysis, we were able to show that two populations of blink frequency existed, corresponding to monomers and dimers, with a homodimer occurrence of 46% for cFos and 87% cJun. This compared well to the dual-color imaging; however, to fully confirm the mixed oligomeric nature of cFos when bound to DNA, we also engineered fluorescent protein fusions with cFos and cJun to analyze FRET activity. A clear FRET signal was observed for cJun, whereas a small increase at the acceptor wavelength was seen for cFos. This suggests that upon homodimerization, the dual-labeled cFos complex affects the quantum yield of the acceptor, which is reduced slightly upon binding DNA. Taken together, these experiments strongly imply that cFos forms a homodimer on DNA in both bulk and single molecule assays. The lower proportion of cFos homodimers may facilitate easier partner swapping with cJun, reducing the energy barrier required for dissociation and reassociation, implying that cFos has evolved to be poorly homodimeric.

These results contrast with previous studies that suggest cFos is limited in its ability to homodimerize (16, 38) and cannot appreciably bind DNA without a partner, *e.g.*, cJun (3). This discrepancy may be due to differing methodologies, for example electrophoretic mobility shift assays may release bound protein due to hydrodynamic forces in the gel’s mesh. Traditional methods such as electrophoretic mobility shift assays, isothermal calorimetry, and size exclusion chromatography typically use high concentrations of protein which may reduce the precision of observations. Our single molecule approaches use a substrate with a large number of binding sites, both cognate and noncognate (17). Such a large number of target sites may increase the probability of detecting a binding event. Studying the binding of cFos to SNP variants of the TRE site would be worthwhile for future studies, as cFos may prefer a different site to the cognate TRE; this has already been performed for the cFos:cJun heterodimer (17). Additionally, the tension experienced by DNA may also be important; *in vivo* DNA is wrapped into chromatin, compacting the genome and inducing tension. DNA tension has been shown to be an important component of protein–DNA interactions and can also be altered by the binding of remodeling factors and the action of polymerases during transcription and replication (39). More recently, tension has been demonstrated to increase off-target binding by Cas9, suggesting that the dynamic tension of the genome may lead to binding which cannot be detected using unconstrained short DNA sequences (40). Previously, the tension on a DNA tightrope was measured as ∼2.2 pN (41), indicating a small but potentially significant alteration to the DNA energy landscape. In support of the hypothesis that the artificial environment of some *in vitro* experiments masks the binding of cFos, it was shown using direct single molecule imaging *in vivo* that cFos homodimers can bind to DNA (27). Despite this, we were able to detect cFos binding to DNA using bulk phase CD spectroscopy, which reports binding through changes in DNA structure. A change in DNA structure could support our hypothesis that tension-induced changes in DNA structure facilitate binding. Single nucleotide variants of CRE DNA oligonucleotides have been shown to produce surprisingly substantial changes to the DNA CD spectrum (19) which may imply that previous studies have used oligonucleotides which were not predisposed to cFos binding due to the composition of the bases chosen to flank the consensus sequence; the physiological significance of the flanking sequences is currently unknown. Further studies are needed to provide a mechanism for these interactions, nonetheless these observations coupled with those of Szalóki *et al*. (27) challenge the traditional belief that cFos is incapable of binding DNA or homodimerizing (3). Indeed, our studies suggest that the proportion of cFos binding to DNA either as monomers or homodimers is significant.

Combining the power of single molecule imaging and bulk phase biochemical assays, we directly show that cFos can bind independently to DNA. This may invoke a new player in the control of gene transcription but requires further stringent *in vivo* study. Although the biological relevance of cFos binding to DNA is yet to be determined, this study provides evidence of DNA-bound cFos monomers and homodimers. Such a perspective is crucial to understanding how these proteins work normally and aberrantly and could provide new targets for inhibition.

## Experimental procedures

### Synthesis of cFos and cJun

Protein sequences from the bZIP region (137–193) from human cFos (UniProt code—P01100) and cJun (252–308) were chemically synthesized and C-terminally biotin-tagged with a preceding gly-ala-pro residue spacer (PeptideSynthetics). Sequences were as follows:

cFos—EEKRRIRRERNKMAAAKCRNRRRELTDTLQAETDQLDEKYALQTEIANLLKEKEKLGAP-Biotin

cJun—RIKAERKRMRNRIAASKCRKRKLERIARLEEKVKTLKAQNYELASTANMLREQVAQLGAP-Biotin

Correct masses were verified by electrospray mass spectrometry. In this study, cFos and cJun refer to the bZIP domains only and do not include transactivation or other domains. *In vitro*–synthesized AP-1 peptides have been used previously and are noted to behave similarly to purified proteins (26, 16, 42). See also supplementary CD spectrum (Fig. S11).

### DNA tightrope substrates and protein-Qdot conjugation

Unmodified bacteriophage Lambda genomic DNA (48.5 kbp, NEB) was used in all assays and contains eight TRE and one CRE consensus sites along its length. Biotinylated proteins were tagged using streptavidin-coated quantum dots (Qdot 655 and Qdot 605; ThermoFisher) by incubating at 100 nM in HSABC (50 mM Tris pH 7.5, 150 mM KCl, and 10 mM MgCl_2_) with 200 nM Qdots (1:2 ratio) for a minimum of 20 min on ice. The proteins were diluted 50-fold in HSABC immediately prior to flowing into the observation chamber. For dual-color homodimer experiments, equimolar Qdots were premixed and then applied to proteins to allow an equal chance of the protein conjugating with either colored Qdot.

### Protein expression and purification

Fluorescent protein–tagged versions of cJun and cFos were also created. These were synthesized by GeneArt (ThermoFisher) to create cJun bZIP fused to mNeonGreen and cFos bZIP fused to mCherry. A hexahistidine tag was inserted at the C terminus to enable purification. The sequences were subcloned into a pCA24N backbone to create two plasmids: pJLJunNG2 and pJLFosCH2. Subsequently, the fluorescent elements of these sequences were swapped using Gibson Assembly to create pJLJunCH and pJLFosNG. For purification, the plasmids were transformed into *E. coli* BL21(DE3), grown at 37 °C to *A*_600_ 0.5 and were induced with 50 μM IPTG. The temperature was dropped to 18 °C, and cells were harvested after 20 h. Following lysozyme and Triton X-100 lysis in the presence of protease inhibitors and DNAse I, the soluble fraction was passed through a nickel affinity spin column, and the protein of interest was eluted with an imidazole gradient. Proteins were buffer exchanged into 50% HSABC/50% glycerol (supplemented with 2.5 mM DTT) and stored at −20 °C.

### Microscopy

Flowcells and DNA tightropes were constructed as described previously (28). In brief, glass beads coated with poly-L-lysine were randomly adhered to a coverslip surface within a flowcell. Lambda DNA was then flowed across the beads to enable suspension of DNA between beads. Fluorescently tagged proteins were then flowed into the flowcells and binding to DNA tightropes imaged. All experiments were performed in HSABC buffer for Qdot conjugates.

Visualization of DNA tightropes was performed using a custom-built oblique angle fluorescence microscope at room temperature (20 ^o^C) as described previously (28). Fluorescence excitation was achieved using an Oxxius 488 nm laser at 5 to 15 mW (depending on fluorophore), guided into the microscope at a subcritical angle to generate far-field illumination. Images were captured using a Hamamatsu ORCA-Flash4.0 V2 sCMOS camera after color splitting through an Optosplit III (Cairn Research Ltd). The three color channels were 500 to 565 nm, 565 to 620 nm, and 620 to 700 nm, and the pixel resolution was measured as 63.2 nm.

### Data analysis

Sixty seconds videos were collected at a frame rate of 10 fps using 1 x 1 binning. A custom ImageJ macro was used to fit kymographs of individual Qdots to a 1D Gaussian distribution (Gaussian Fit Extra: available from https://github.com/Kad-Lab/ImageJ). The kymograph fitting algorithm was unbiased, and therefore during a blink, it would attempt to fit background fluorescence. These fits were consistently poor (R^2^ <0.7) compared with >0.9 for accurate fitting in the presence of a Qdot signal. This provided an excellent means to threshold filter the fits and determine number and duration of blinks.

To fit the Qdot blinking data, we used a combined Poisson approach. Two Poisson relationships were fitted using Microsoft Excel (GRG engine), simultaneously to both the cJun and cFos data. Firstly, the monomer blinking probability is given by the expected value for monomer blinks/kymograph (*bl*):

(1) pmon=bln⋅e−bln!

Where *n* is the number of blinks. By linking the expected value for the dimer population to the blinking probability of the monomer, it was possible to reduce the number of fitted parameters. The expected number of dimer blinks (*bl*_*dim*_) was calculated from the expected value of monomer blinks by conversion to blink rate per second using the movie duration (*dur*). This value is squared because the dimer blinking rate is the square of the monomer and then returned to an expected value by multiplying by *dur*:

(2) bldim=(bldur)2·dur

The expected dimer blinking value was then used as in Equation 1 to calculate the probability distribution for dimer blinks (*p*_*dim*_). The probability distributions were normalized to the total number of blinks *via* an amplitude term (*α*) which summed to one between monomer and dimer:

(3) $$p_{blink}=\alpha \text{·}p_{mon}+\left(1-\alpha \right)\text{·}p_{dim}$$

This calculation was performed on-the-fly during minimization of the sum of square differences for *p*_*blink*_. Since the same Qdots were used for both cFos and cJun, the same expected blinking values could be used to fit both these datasets simultaneously. Therefore, only the amplitudes and the expected monomer value of blinks/kymograph were allowed to vary during the fit.

### FRET assay

cJun-mNeonGreen (JNG), cJun-mCherry (JCH), cFos-mNeonGreen (FNG), and cFos-mCherry (FCH) were added to a 96-well plate at 400 nM each in HSABC buffer supplemented with 2.5 mM DTT. 500 nM of a short TRE-containing DNA duplex was also added to all wells: sequence 5′GTCAGTCAG**TGACTCA**ATCGGTCA (Eurofins Genomics). Plates were incubated at room temperature for 10 min and then analyzed using a Spectramax ID5 Plate Reader. Fluorescence spectra were obtained using an excitation scan between 450 and 650 nm with a step size of 10 nm and a fixed emission of 700 nm, necessary to enable the full excitation spectrum to be scanned. Datasets represent the average of three independent repeats.

### Circular dichroism

An Applied Photophysics Chirascan was used for CD measurements, with a 200 μl sample in a 1 mm path length CD cell. Protein/DNA samples were suspended in 150 mM potassium phosphate, 150 mM potassium fluoride, and 5 mM tris(2-carboxyethyl)phosphine at pH 7.4. Spectra were collected between 190 and 320 nm with a bandwidth of 1 nm, sampled at 0.5 nm s^−1^. For each sample, three scans were collected and averaged. The following 24 bp double-stranded oligonucleotide sequences were used, TRE: 5′GTCAGTCAG**TGACTCA**ATCGGTCA, control non-TRE: 5′CCTGCGTAGTTCCATAAGGATAGC (Sigma) (43).

## Data availability

Data are available upon request.

## Supporting information

This article contains supporting information (16).

## Conflict of interest

The authors declare that they have no conflicts of interest with the contents of this article.

## Supporting information

SUPPLEMENTARY MATERIAL_revised_FINAL
