# Supplementary material for: In vitro single molecule and bulk phase studies reveal the AP-1 transcription factor cFos binds to DNA without its partner cJun
Source: J Biol Chem. 2022 Jul 1;298(8):102229. doi: 10.1016/j.jbc.2022.102229 (PMC9364023; doi:10.1016/j.jbc.2022.102229)
Supplement: SUPPLEMENTARY MATERIAL_revised_FINAL [file mmc1.pdf]

SUPPLEMENTARY MATERIAL: *In vitro* single molecule and bulk phase studies reveal the AP-1 transcription factor cFos binds to DNA without its partner cJun

James T. Leech[1], Andrew Brennan[2], Nicola A. Don[1], Jody M. Mason[2] and Neil M. Kad[1]

[1]School of Biological Sciences, University of Kent, Canterbury, CT2 7NH, UK. [2] Department of Biology & Biochemistry, University of Bath, Bath, BA2 7AY, UK.

Contents:

1. **Figure S1-S9: Excitation spectrum FRET explained**
2. **Figure S10: Control Excitation FRET spectrum**
3. **Figure S11: Full CD Spectra for biotinylated chemically synthesized cJun proteins**

1. Excitation spectrum FRET explained

To determine the extent of Förster resonance energy transfer from mNeonGreen (NG) to mCherry (CH) we took excitation spectra where the emission wavelength was fixed at 700 nm and the excitation was scanned from 450 to 650 nm. The emission maximum of 700 nm was chosen to compromise the large bandwidth of the fluorescence plate reader (Spectramax ID5), and the emission spectrum of mCH (Figure S1). The excitation and emission spectra shown were obtained from FPBase.org. All other spectra were measured.

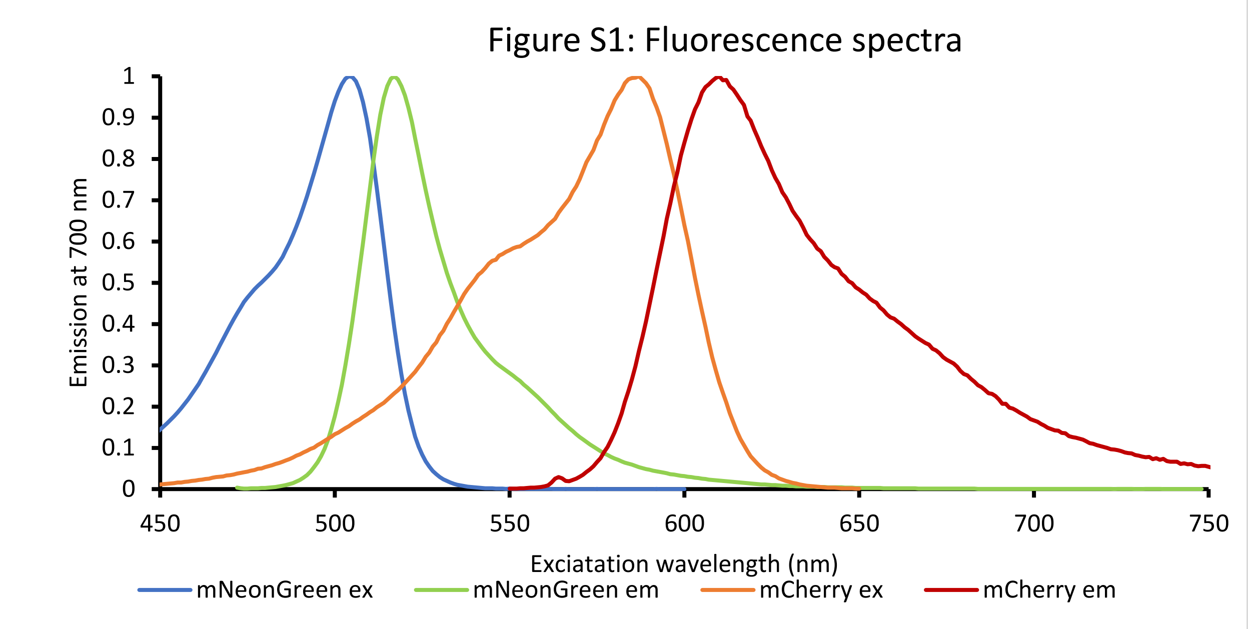

The protein species used were cJun-CH, cJun-NG and cFos-CH, cFos-NG, and these were always mixed as homodimers never as cJun and cFos.

Starting with cJun, Figure S2 shows the excitation spectra +/- DNA of cJun-CH only:

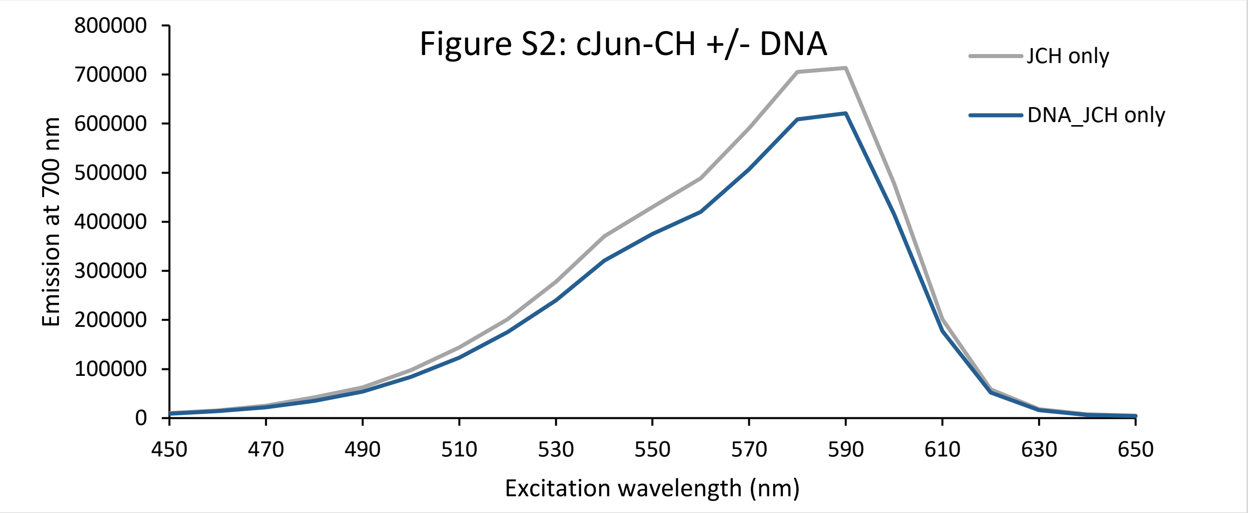

The addition of DNA leads to a small but clear change in the excitation maximum of CH. Similarly, there is a reduction in the observed intensity for NG, with an emission maximum at 700 nm. These spectra provide controls for the mixture experiment.

Then with Jun-NG only:

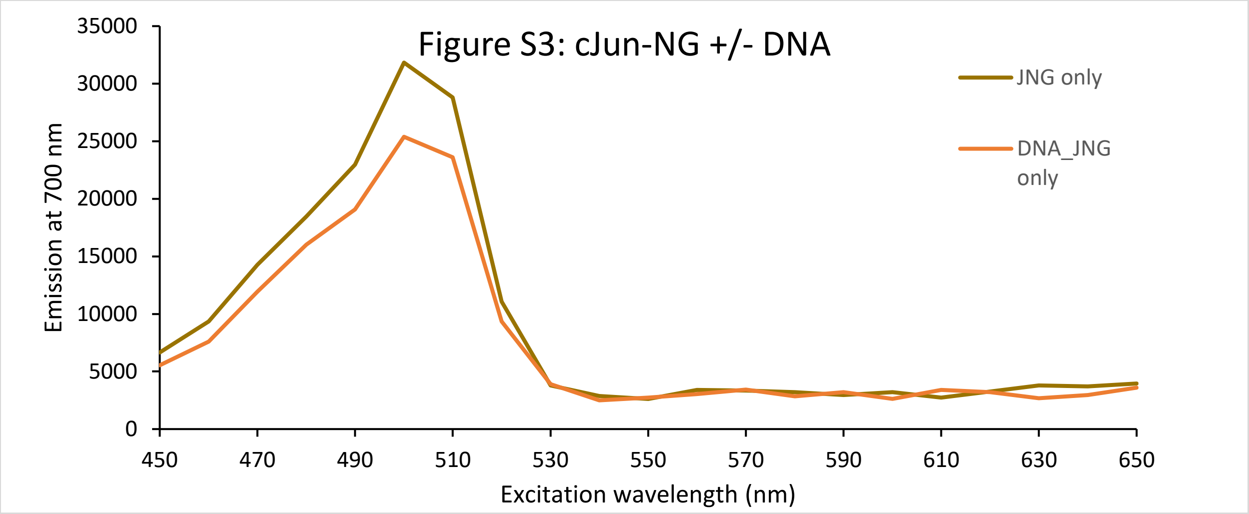

Figure S4, shows the resulting spectra of cJun-NG mixed with Jun-CH +/-DNA.

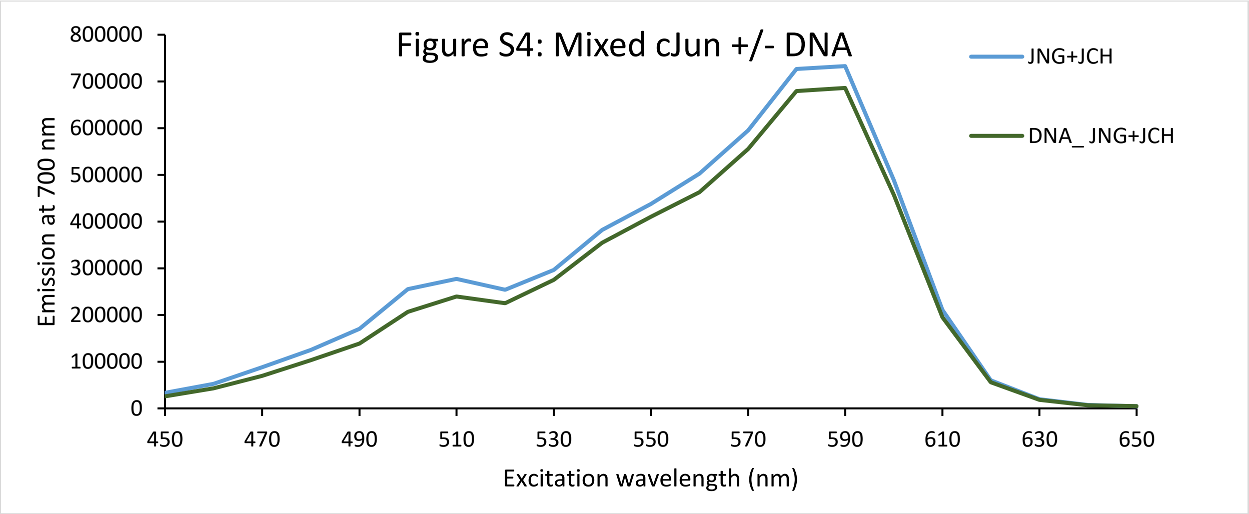

A clear contribution at ~500 nm, the excitation maximum for NG is observed. To make this clearer we subtracted the for the proteins together vs the linear sum of their component spectra (Figure S5).

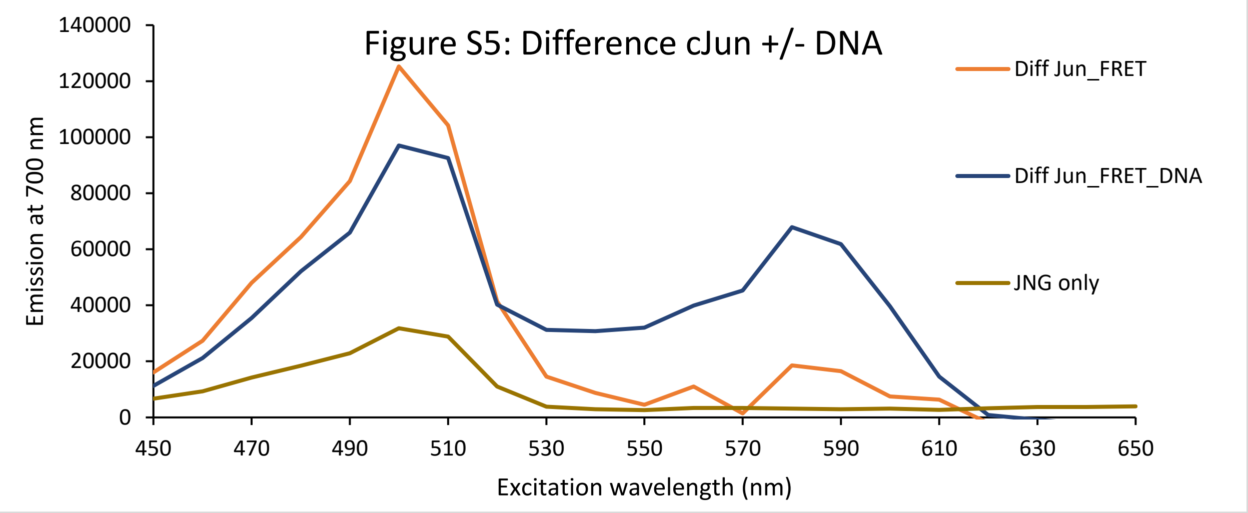

The clear large peak in the absence of DNA indicates energy transfer to CH from NG (compare with Jun-NG alone (JNG)). In the presence of DNA, it is less efficient most likely due to a conformational change in the dimer leading to a loss of FRET efficiency.

The equivalent spectra to Figure S2 for cFos is shown in Figure S6. Here, no effect on the fluorescence of CH is seen in the presence of DNA relative to its absence.

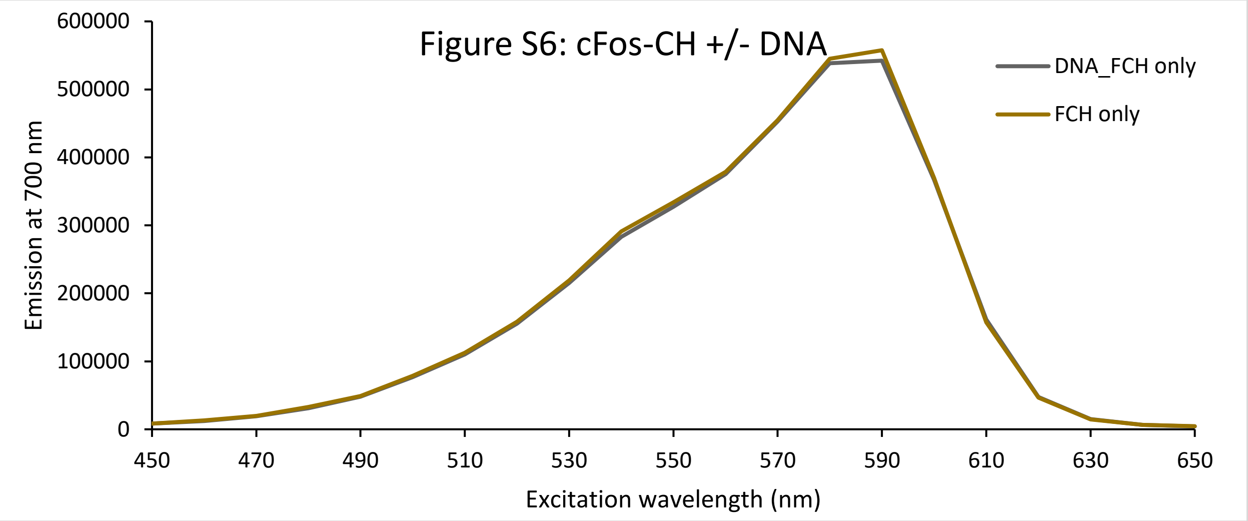

This is also true for cFos-NG +/- DNA (Figure S7).

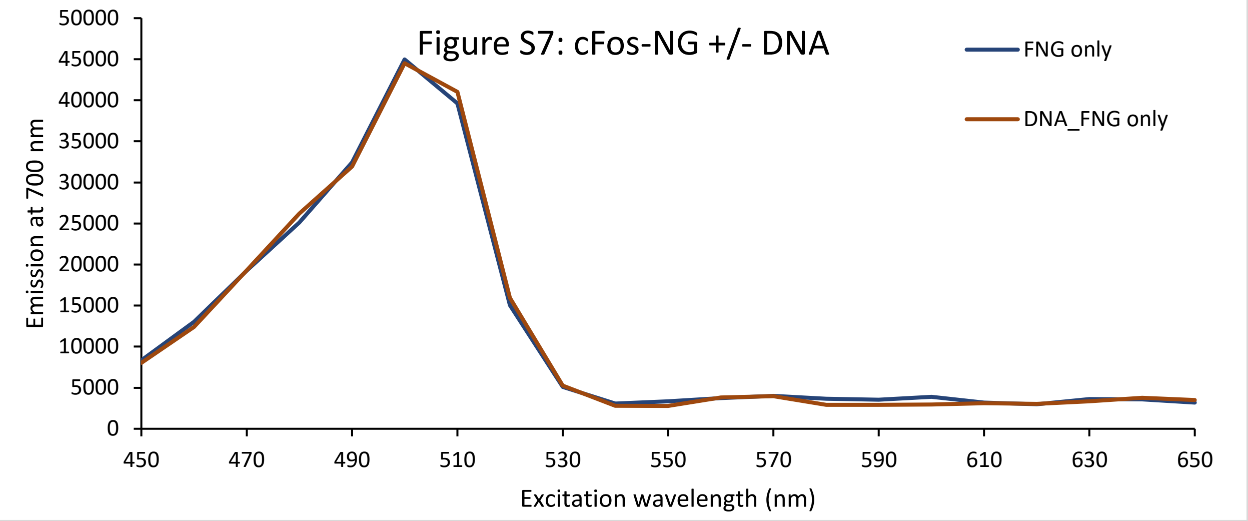

When the two differentially labelled species are mixed together (Figure S8), a small change in the fluorescence excitation for CH is seen.

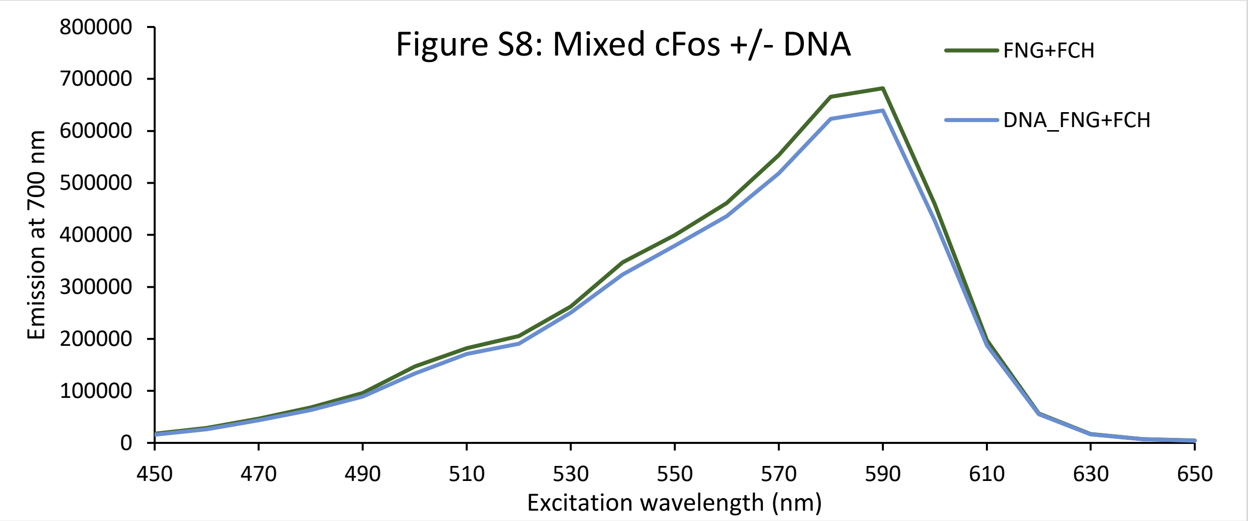

When shown as difference spectra based on subtracting the proteins together vs the linear sum of their component spectra and this time adding Fos-NG as a reference the magnitude of the change in the presence of DNA is clearly seen. Furthermore, there is a reduction in the fluorescence contribution of NG to the signal at 700 nm. Because these changes are only apparent when the differentially labelled proteins are mixed this indicates that the signal derives from an interaction between the fluorophores. The origin of this change is not provided in the analysis, however, these data clearly indicate that the two fluorophores are close enough together to interact, therefore suggesting a cFos dimer can form and the structure of this dimer is altered by the presence of DNA.

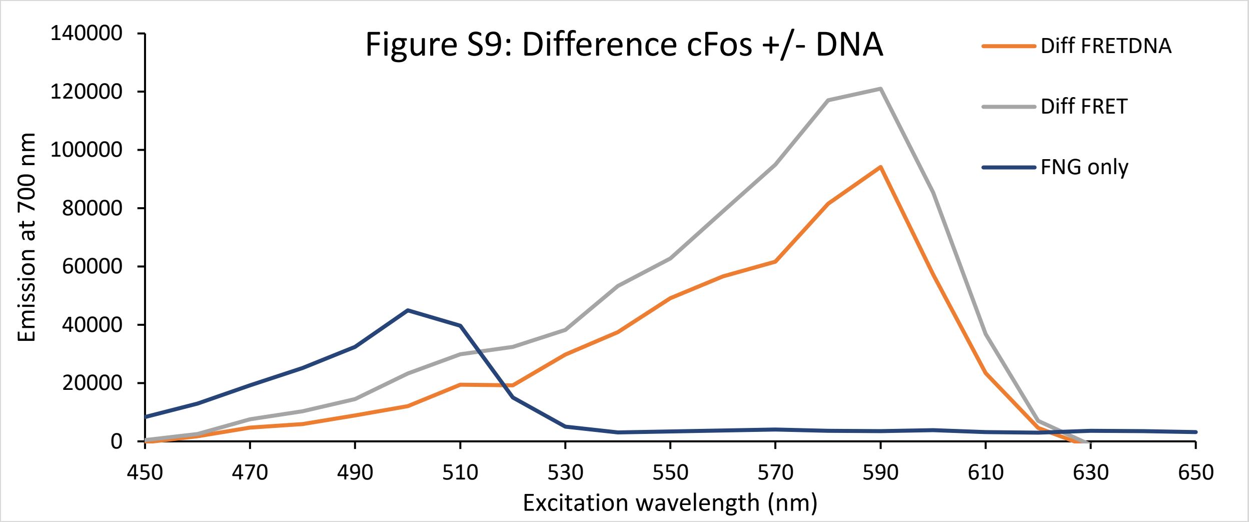

Figure S5 (Figure 3B main manuscript) shows no 590 nm peak for cJun; we suggest that the conformation of the dimeric forms of cJun and cFos are substantially different which reconciles Figure S5 with Figure S9. A comparison of the published structures of cJun homodimers (5T01) and cFos-cJun heterodimers (1FOS) reveal the C-termini are in different positions, therefore it is not surprising that cFos homodimers could have different positions of the fluorophores that explains the different spectra. Furthermore, the cJun FRET spectra show a 590 nm peak in the presence of DNA (figure S5) suggesting a conformational effect upon DNA binding, another demonstration of the powerful approach of excitation spectra FRET.

2. Figure S10: Control Excitation FRET spectrum

To ensure that the observed changes in emission at 700 nm were due to the interaction of cFos and not the fluorophores themselves interacting, we examined the spectrum of UvrA C-terminally fused with mNeonGreen (UvrANG)and cFos-mCherry. The excitation spectrum in Figure S10 shows no significant change in the emission at 700 nm when these proteins were mixed at the same concentrations used in the main manuscript (400 nM). The UvrA-mNG fusion was expressed and purified following the same procedure as cFos and cJun (materials and methods – main manuscript).

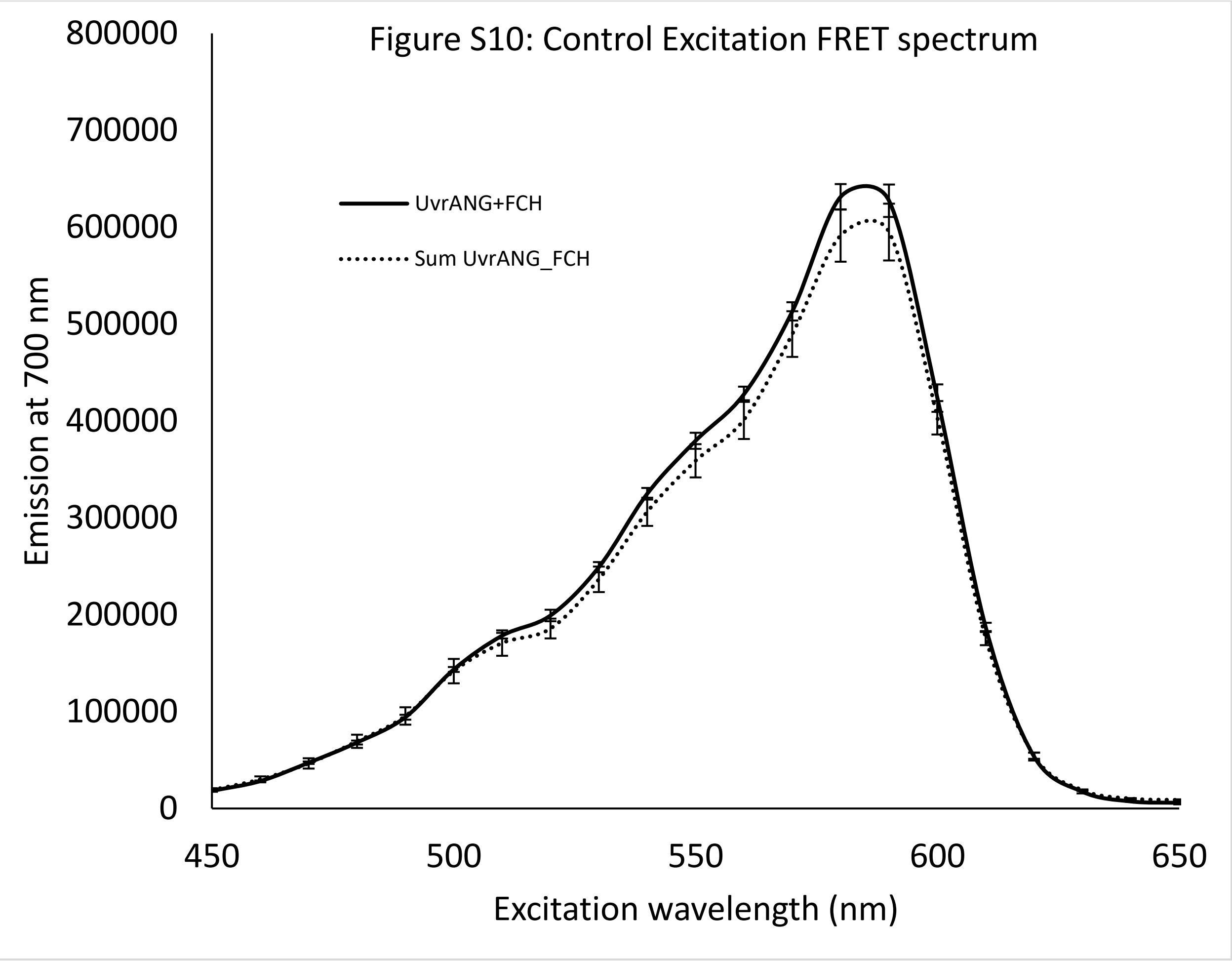

Figure S10: *Fluorescence excitation sweep with a fixed emission of 700 nm with cFos-mCherry mixed with UvrA-mNeonGreen. The data shows no significant change in fluorescence when these two non-interacting partners were added together, suggesting an absence of energy transfer effects. This also provides evidence that the cFos-mNeonGreen (FNG) – cFos-mCherry (FCH) fluorescent changes are specific and significant.*

3. Figure S11: Full CD Spectra for biotinylated chemically synthesized cJun proteins

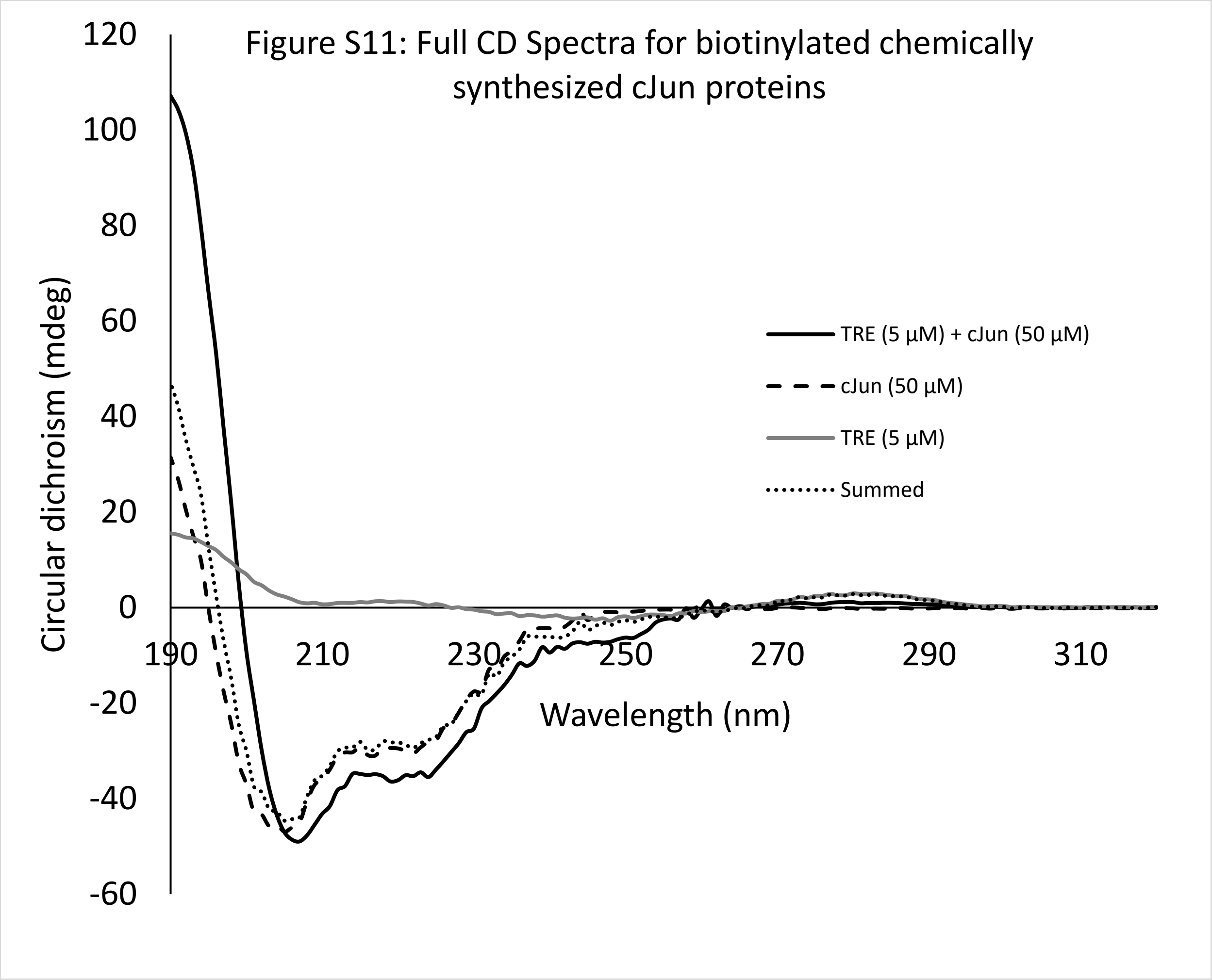

Figure S11: *Complete CD spectra for biotinylated cJun binding to TRE DNA. Values are comparable to those observed in Mason* et al*. (2006) indicating that the biotin tag did not affect the helicity of the peptide.*
