# Supplementary material for: In vitro single molecule and bulk phase studies reveal the AP-1 transcription factor cFos binds to DNA without its partner cJun
Source: J Biol Chem. 2022 Jul 1;298(8):102229. doi: 10.1016/j.jbc.2022.102229 (PMC9364023; doi:10.1016/j.jbc.2022.102229)
Supplement: Supplementary movies [file mmc2.pptx]

## Slide 1
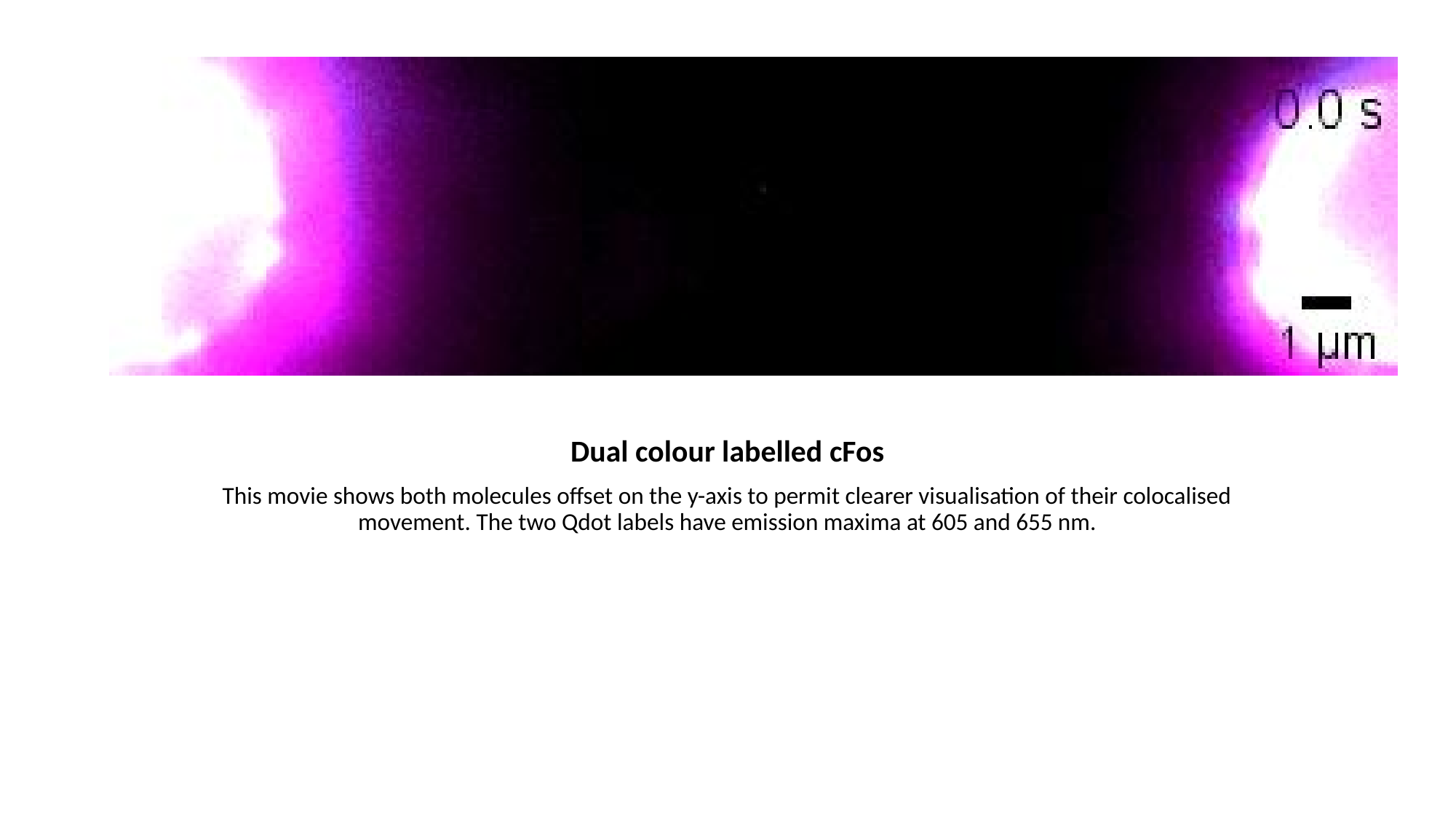

Dual colour labelled cFos
This movie shows both molecules offset on the y-axis to permit clearer visualisation of their colocalised movement. The two Qdot labels have emission maxima at 605 and 655 nm.

## Slide 2
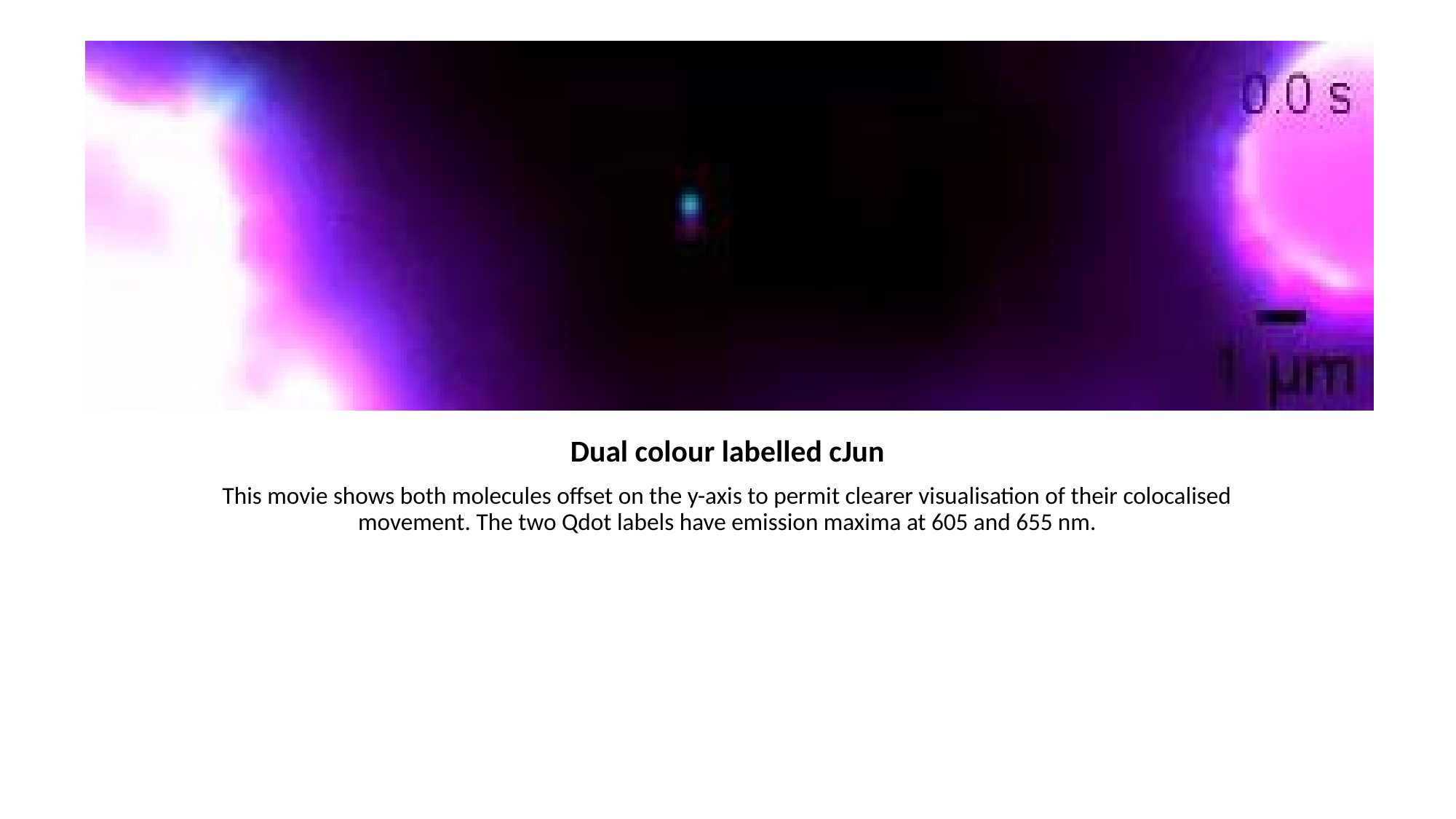

Dual colour labelled cJun
This movie shows both molecules offset on the y-axis to permit clearer visualisation of their colocalised movement. The two Qdot labels have emission maxima at 605 and 655 nm.
